# Association Between Household Food Insecurity and Malnutrition Among Children Attending Anganwadi Centres in Rural Bengaluru: A Community-Based Cross-Sectional Study

**DOI:** 10.7759/cureus.47007

**Published:** 2023-10-13

**Authors:** Hamsa L, Ranganath T S, Raksha R Nayak, Pradeep Kumar N G

**Affiliations:** 1 Preventive and Social Medicine, Bangalore Medical College and Research Institute, Karnataka, Bengaluru, IND; 2 Community Medicine, Bangalore Medical College and Research Institute, Bengaluru, IND

**Keywords:** anganwadi children, rural, household food insecurity, underweight, wasting, stunting

## Abstract

Background and objectives: Malnutrition is a universal problem that has many forms. It affects all geographies, all age groups, and rich and poor people.The link between food insecurity and the health of populations has been established. Malnutrition commonly affects all groups in a community, but infants and young children are the most vulnerable because of their high requirements for growth and development. Hence, this study is taken up to assess existing household food insecurity, nutritional status, and various factors influencing the same among preschool children.

Material and methods: This was a cross-sectional study conducted in Anganwadis in the rural field practice area attached to Bangalore Medical College and Research Institute (BMCRI), Bengaluru. Based on the probability proportional to size (PPS) sampling method, 500 Anganwadi children aged three to six years were included in the study from nine Integrated Child Development Scheme (ICDS) blocks in Nelamangala, Bengaluru. Data were collected using a pre-validated, semi-structured questionnaire.

Results: Among 500 study participants, 274 (54.8%) were boys and 226 (45.2%) were girls. Moderate underweight was seen in 13.87% of boys and 28.32% of girls. Severe underweight was seen in 6.57% of boys and 4.4% of girls. Moderate stunting was seen in 6.2% of boys and 21.68% among girls. Severe stunting was seen in 1.44% of boys and 0.88% of girls. Moderate wasting was seen in 12.41% of boys and 16.81% of girls. Severe wasting was seen in 2.19% of boys and 0.88% of girls. Mild food insecurity was seen in 11.65% of households, and moderate food insecurity was seen in 5.2% of households. There was a significant association between wasting and household food insecurity (p < 0.05), stunting, and household food insecurity (p < 0.05). There was a significant correlation between age and stunting, gender and stunting, and gender and underweight. On regression analysis, moderate food insecurity was 2.08 times higher and significantly associated with stunting.

Conclusion: The prevalence of malnutrition was less than the NFHS 5 statistics in this study. Regular monitoring and analysis of food insecurity and malnutrition among children and women need to be done at the national, state, and regional levels.

## Introduction

Malnutrition is a universal problem that has many forms. No nation is unaffected. All types of malnutrition are linked to a range of illnesses and a greater mortality rate. Over 45% of deaths among children under five are attributed to undernutrition, mostly in low- and middle-income nations [1]. Malnutrition is a social and economic issue that hinders global development and has unacceptable effects on people. According to estimates, the cost of malnutrition in all its forms to society might reach US$3.5 trillion annually [2].

Childhood malnutrition is influenced by biological, behavioural, and environmental factors. Poor nutrition can lead to stunted growth, which is associated with impaired cognitive ability and reduced school and work performance [3,4].

Globally, the prevalence of stunting is around 149.2 million, or 22% of under-5 children. The prevalence of wasting is estimated at approximately 45.4 million, or 6.5%, of children under five years of age. Around 45% of deaths among children under five years of age are linked to undernutrition. These mostly occur in low- and middle-income countries [5].

India is one of the nations with severe child undernutrition, and it also contributes significantly to child mortality in India. One in three malnourished children worldwide, according to UNICEF, is Indian [1,6]. On comparing the NFHS-4 and NFHS-5 nutritional status of rural under-5 children in India, the prevalence of underweight, stunting, and wasting according to NFHS-4 was 38.3%, 41.2%, and 22%, respectively. The prevalence of underweight, stunting, and wasting, according to the NFHS-5, was 33.8%, 37.3%, and 19.5%, respectively. Similarly, in Karnataka, the prevalence of being underweight, stunting, and wasting, according to the NFHS-4, was 37.7%, 38.5%, and 27%, respectively. The prevalence of underweight, stunting, and wasting, according to the NFHS-5, was 34.9%, 37.2%, and 20.1%, respectively [7,8].

There are various risk factors associated with malnutrition: socioeconomic status, family size, literacy, household food insecurity, etc. [9]. Food security “exists when all people at all times have physical and economic access to sufficient and nutritious food to meet their dietary needs and food preferences for an active and healthy life” [1].

Historically, in India, efforts have been made to achieve food security among the population through the public distribution system. The integrated Child Development Services scheme was launched way back in 1975 and is one of the largest and most unique programmes for early childhood development, along with many other national nutritional programmes [10]. The National Food Security Act 2013 has been passed to provide an adequate quantity of quality food at affordable prices. Even with all these efforts, India is home to one in three malnourished children in the world, according to UNICEF. Hence, this study was conducted to assess existing household food insecurity, nutritional status, and various factors influencing the same among preschool children.

## Materials and methods

Objectives

To assess the malnutrition of the children attending Anganwadi centres in rural Bengaluru and to determine the association between household food insecurity and malnutrition.

Methodology

This is a community-based cross-sectional study from February 2021 to July 2022 in the Nelamangala rural field practice area attached to Bangalore Medical College and Research Institute (BMCRI). The study population includes children attending Anganwadi in the rural field practice area attached to BMCRI. Based on a previous study conducted by Pathak et al. [11], by considering the prevalence of stunting, which is 36.4% with a confidence interval of 95%, the sample size came to around 500. After obtaining consent from the institutional ethical committee, probability proportional to size (PPS) sampling and simple random sampling techniques were used to recruit the study participants. The inclusion criteria were children three to six years of age attending Anganwadis, their parents or guardians, and a parent/guardian willing to give informed consent. The exclusion criteria were children who were absent due to causes other than illness. A pre-tested, pre-validated, semi-structured questionnaire and the Household Food Insecurity Assessment Scale (HFIAS) [12].

Data Collection

After obtaining ethical clearance from the Institutional Ethical Committee of Bangalore Medical College and Research Institute (IRB no: BMCRI/PG/131/2020-21), permission was sought from the Child Development Project Officer (CDPO) and Taluk Health Officer (THO) of Nelamangala block. There are a total of nine ICDS blocks in Nelamangala, with 2100 Anganwadi children. Each ICDS block has seven to nine Anganwadis. Each Anganwadi had 10-30 children. By considering the average, we get 20 children in each Anganwadi centre. In each ICDS block, there were around 160 Anganwadi children. Thirty percent of the children in each Anganwadi centre were selected using a simple random sampling method based on the lottery method. The population comes to around 480, which was rounded up to 500 to meet the target sample size.

In the Anganwadis, the first contact person was the Anganwadi teacher. They have explained the purpose of the study, then, with the help of an Anganwadi helper, contacted the parents of the children, explained the study, obtained informed consent for the interview, and did anthropometric measurements of their children. Listed the number of children who were present in the Anganwadis on the day of data collection. Then we measured the anthropometry of the children and interviewed the children’s parents/guardians.

Statistical analysis

The data collected were analyzed using SPSS version 21.0 (IBM SPSS, Armonk, NY). The data collected were entered in Microsoft Excel (Microsoft® Corp., Redmond, WA) and analyzed using SPSS version 20.0. Sociodemographic data were presented using descriptive statistics, namely frequency (n), percentage (%), mean, standard deviation, median, and interquartile range, wherever applicable. The chi-square test was used to determine the association between the categorical variable and the dependent variable. Bivariate analysis was to assess the correlation between nutritional status and demographic variables. Logistic regression analysis was done to assess the odds ratio between anthropometric measures and household food insecurity. p < 0.05 was considered statistically significant. Data were presented in the form of tables, figures, and graphs wherever necessary.

## Results

A total of 500 children participated in this study, out of which 274 (54.80%) were boys and 226 (54.2%) were girls. The mean age of the children was 4.1, with a standard deviation of 0.89 years. 377 (75.4%) children were Hindus, and 123 (24.6%) were Muslims. The majority of them, 347 (69.4%), belonged to the nuclear family. Among the study participants, 284 (56.8%) belong to the below poverty line (BPL), and 216 (43.2%) belong to the above poverty line (APL). Other sociodemographic variables are mentioned in Table 1.

**Table 1:** Sociodemographic variables The data have been represented as frequency(n) and percentage (%)

Variable	Frequency (n)	Percentage (%)
Age (in years)		
3–4	320	64.00%
4–5	125	24.00%
5–6	55	12.00%
Religion		
Hindu	377	75.40%
Muslim	123	24.60%
Type of family		
Joint	38	7.60%
Nuclear	347	69.40%
Three generation	115	23.00%
Education of mother		
Graduate	15	3.00%
Post-high school diploma	5	1.00%
High school	283	56.60%
Middle school	38	7.60%
Primary school	98	19.60%
Illiterate	61	12.20%
Occupation of the head of the family		
Professional	1	0.20%
Semi-professional	2	0.40%
Clerical, shop owner	52	10.40%
Skilled worker	254	50.80%
Semiskilled worker	62	12.40%
Unskilled worker	129	25.80%
Socio economic status		
Upper class	1	0.20%
Upper middle class	45	9.00%
Middle class	196	39.20%
Lower middle class	191	38.20%
Lower class	67	13.40%

Food security was reported in 235 (85.77%) of households with a male child and 182 (80.53%) of households with a female child. In households with male and female children, there were 30 (10.95%) and 28 (12.23%) cases of mild food insecurity, respectively. As demonstrated in Table 2, homes with male and female children had 9 (3.28%) and 16 (7.08%) moderate levels of food insecurity, respectively. There was no statistically significant difference between genders with respect to various categories of household food insecurity (p>0.05).

**Table 2:** Distribution of household food insecurity status (n=500) The data have been represented as N, % The p-value is not statistically significant at p<0.05

Food security status	Boys		Girls		P-value
	n	%	n	%	
Food-secure	235	85.77%	182	80.53%	χ^2^ = 4.20, p = 0.122
Mild food insecure	30	10.95%	28	12.39%	
Moderate food insecure	9	3.28%	16	7.08%	
Severe food insecure	0	0.00%	0	0.00%	
Total	274	100.00%	226	100.00%	

**Figure 1:**
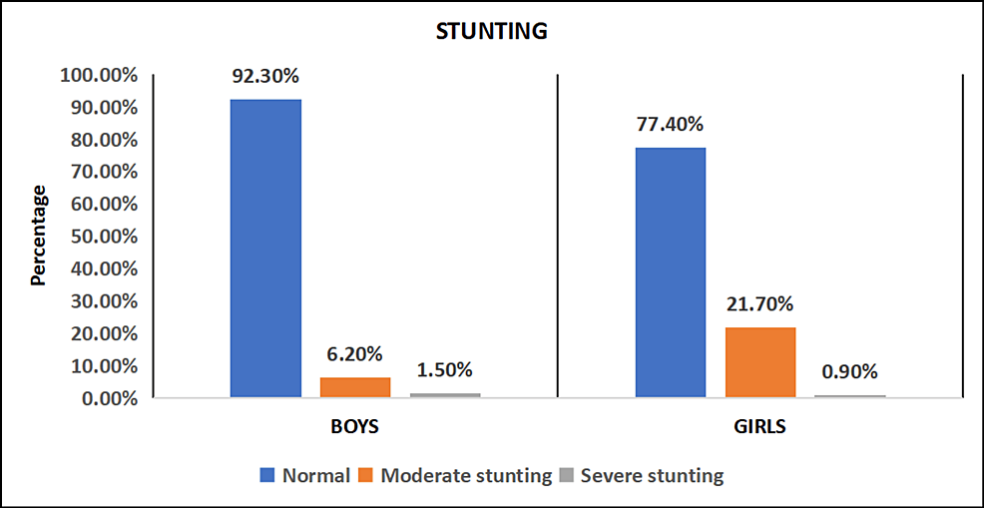
Distribution of study subjects based on stunting (n = 500)

The prevalence of stunting (height for age) was assessed, and we found that 253 (92.34%) of boys and 175 (77.43%) of girls have normal height for age. Moderate stunting was seen in 17 (6.20%) boys and 49 (21.68%) girls. Severe stunting was seen in 4 (1.46%) boys and 2 (0.88%) girls (Figure 1).

**Figure 2:**
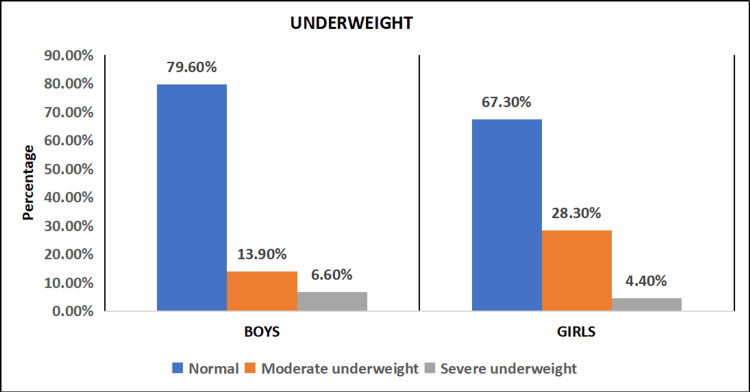
Distribution of study subjects based on underweight (n = 500)

The prevalence of underweight (weight for age) revealed that 218 (79.56%) of boys and 152 (67.26%) of girls have normal weight for age. There were 64 (28.32%) girls and 38 (13.87%) boys who were moderately underweight. Severe underweight was seen in 18(6.57%) among boys and 10(4.42%) among girls (Figure 2).

**Figure 3:**
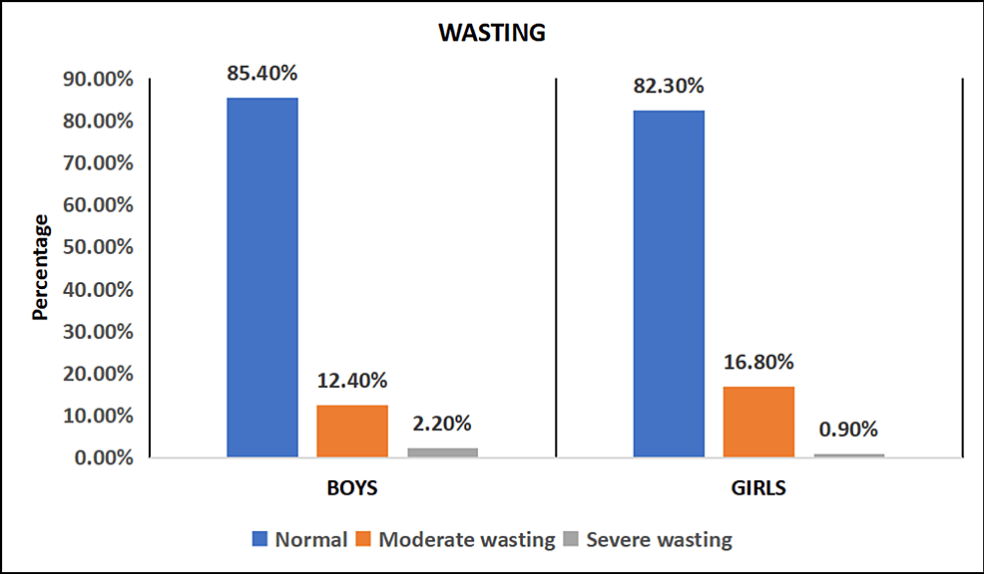
Distribution of study subjects based on wasting (n = 500)

The prevalence of wasting (weight for height) was assessed, and it was discovered that 234 (85.40%) of boys and 186 (82.30%) of girls have normal weight for age. Moderate wasting was observed in 34 (12.41%) of the boys and 38 (16.81%) of the females. Severe wasting was observed in 6 (2.19%) of the boys and 2 (0.88%) of the girls (Figure 3). The overall comparison of stunting, underweight and wasting is mentioned in Table 3.

**Table 3:** Distribution based on stunting, wasting, and underweight (n=500) χ2 = chi-square value *<2 z-score and > −3 z-score **−3 z-score ***The p-value is considered statistically significant at p < 0.001

	Boys		Girls		p-value
	n	%	n	%	
Height for age (stunting)					
Normal	253	92.34%	175	77.43%	χ^2^ = 26.03, p = < 0.0001***
Moderately stunted*	17	6.20%	49	21.68%	
Severely stunted**	4	1.46%	2	0.88%	
Total	274	100.00%	226	100.0%	
Weight for age (underweight)					
Normal	218	79.56%	152	67.26%	χ^2^ = 16.23, p = < 0.0001***
Moderately underweight*	38	13.87%	64	28.32%	
Severely underweight**	18	6.57%	10	4.42%	
Total	274	100.00%	226	100.00%	
Weight for height (wasting)					
Normal	234	85.40%	186	82.30%	χ^2^ = 3.12, p = 0.21
Moderate wasting*	34	12.41%	38	16.81%	
Severe wasting**	6	2.19%	2	0.88%	
Total	274	100.00%	226	100.0%	

Among food-secure households, 79 (18.94%) were moderately underweight, and 20 (4.80%) were severely underweight. Among mild food-insecure households, 14 (24.14%) were moderately underweight and 5 (8.62%) were severely underweight. Among moderately food-insecure households, 9 (36.0%) were moderately underweight and 3 (12.00%) were severely underweight. The association between household food insecurity and underweight was not statistically significant (p > 0.05).

Among food-secure households, 43 (10.31%) had moderate stunting and 4 (0.96%) had severe stunting. Among mild food-insecure households, 12 (20.69%) had moderate stunting and 2 (3.45%) had severe stunting. Among moderately food-insecure households, 11 (44.0%) had moderate stunting and 0 (0%) had severe stunting. The association between household food insecurity and stunting was statistically significant (p < 0.05).

Among food-secure households, 48 (11.51%) had moderate wasting and 6 (1.44%) had severe wasting. Among mild food-insecure households, 13 (22.41%) had moderate wasting and 1 (4.00%) had severe wasting. Among moderately food-insecure households, 11 (44.0%) had moderate wasting and 1 (4.00%) had severe wasting. The association between household food insecurity and wasting was statistically significant (p < 0.05).

On applying Pearson’s correlation test, there was a negative correlation between underweight and food-secure households, stunting and food-secure households, and wasting and food-secure households. There was a positive correlation between underweight and moderate food insecurity, stunting and moderate food insecurity, and wasting and moderate food insecurity (Table 4).

**Table 4:** Correlation between nutritional status and food insecurity (n=500) *p-value is considered statistically significant at p<0.05

	Food-secure	Mild insecurity	Moderate insecurity
Underweight			
Pearson correlation	−0.116	0.062	0.114
p-value	0.009*	0.167	0.011*
Stunting			
Pearson correlation	−0.188	0.110	0.165
p-value	0.0001*	0.014	0.0001*
Wasting			
Pearson correlation	−0.168	0.071	0.188
p-value	0.0001*	0.112	0.0001*

On logistic regression analysis, moderate food insecurity has a significant odds ratio with stunting. The stunting rate was 2.08 times higher among moderately food-insecure households (Table 5).

**Table 5:** Logistic regression analysis between household food insecurity and malnutrition AOR: adjusted odds ratio *P-value is considered statistically significant at p<0.05

	Stunting		Wasting		Underweight	
	AOR	p-value	AOR	p-value	AOR	p-value
Food-secure	1	-	1	-	1	-
Mild food insecurity	0.90	>0.05	0.71	>0.05	0.54	>0.05
Moderate food insecurity	2.08	<0.05*	1.76	>0.05	1.4	>0.05

## Discussion

Socio-demographic characteristics

In our study involving 500 preschool children, the sex ratio revealed a lower number of girls [226 (45.20%)] compared to boys [274 (54.80%)], which is notably below the national child sex ratio of 1037 females for every 1000 males in rural areas. This disparity contrasts with prior research studies conducted by Gubert et al. [13], Pathak et al. [11], and Abedi et al. [14], which reported varying proportions of male and female children. Regarding religious composition, the majority in our study were Hindus [377 (75.40%)], with 123 (24.60%) being Muslims. This differs from the findings in the studies by Pathak et al. [11] and Gubert et al. [13], which had varying religious distributions. In terms of family structure, most households in our study were nuclear families [347 (69.40%)], followed by three-generation families [115 (23.00%)], and the remaining were joint families. These figures were somewhat consistent with other studies by Pathak et al. [11] and Das et al. [15]. Regarding socio-economic status, most households fell into the middle-class category [196 (39.20%)], followed by the lower-middle class [191 (38.20%)] as per the Modified B.G. Prasad classification. This distribution differed from findings in other studies by Abedi et al. [14], Pathak et al. [11], Kundapur et al. [16], and Gubert et al. [13], where lower socioeconomic classes predominated.

Association between household food insecurity and nutrition status

In our study, among children from food-secure households, 318 (76.25%) had a normal weight for their age, 79 (18.94%) were moderately underweight, and 20 (4.80%) were severely underweight. Among mildly food-insecure households, 39 (67.24%) had a normal weight, 14 (24.14%) were moderately underweight, and 5 (8.62%) were severely underweight. In moderately food-insecure households, 13 (52%) had a normal weight, 9 (36%), were moderately underweight, and 3 (12%) were severely underweight. Interestingly, the study did not find a statistically significant association between household food insecurity and underweight (p > 0.001). Comparing our study findings to other research, Pathak et al. [11] reported varying degrees of underweight among food-secure and food-insecure households, with a higher prevalence in the latter. Das and Krishna [16] found a higher overall underweight prevalence among food-insecure household children. Gubert et al. [13] also observed a higher prevalence of underweight among food-insecure children. In contrast, a study by Berra [17] in Ethiopia found lower prevalence rates of underweight across different food security levels. Overall, our study suggests a complex relationship between household food security and child underweight, with variations in findings compared to other research, highlighting the need for further investigation.

In our study, among food-secure households, 370 (88.73%) of children had normal height-for-age, 43 (10.31%) had moderate stunting, and 4 (0.96%) had severe stunting. In mildly food-insecure households, 44 (75.66%) had normal height, 12 (20.69%) had moderate stunting, and 2 (3.45%) had severe stunting. Among moderately food-insecure households, 14 (56%) had normal height, with 11 (44%) experiencing moderate stunting and no severe stunting cases. The association between household food insecurity and stunting was statistically significant (p < 0.001). Comparatively, other studies, such as Pathak et al. [11], Das et al. [16], Gubert et al. [13], and Abedi et al. [14], also noted a higher prevalence of stunting among food-insecure households. However, Berra's [17] study in Ethiopia reported lower stunting rates in food-secure households, highlighting regional differences in this issue. Therefore, our study emphasizes the strong link between household food insecurity and child stunting, with varying prevalence rates compared to other research, underscoring the importance of addressing food security for children's growth and development.

In our study, among food-secure households, 363 (87.05%) of children had normal weight for height, 48 (11.51%) had moderate wasting, and 6 (1.44%) had severe wasting. In mildly food-insecure households, 44 (75.66%) had a normal weight for height, 13 (22.41%) had moderate wasting, and 1 (4%) had severe wasting. Among moderately food-insecure households, 13 (52%) had a normal weight for height, 11 (44%) experienced moderate wasting, and 1 (4%) had severe wasting. The association between household food insecurity and wasting was statistically significant (p < 0.001). Comparatively, other studies by Pathak et al. [11], Das et al. [16], Gubert et al. [13], and Abedi et al. [14] also noted a higher prevalence of wasting among food-insecure households. However, Berra's [17] study in Ethiopia reported slightly higher wasting rates in moderately food-insecure households compared to those that were mildly food-insecure. Our study highlights a clear link between household food insecurity and child wasting, with varying prevalence rates compared to other research, emphasizing the crucial role of food security in promoting healthy growth and development among children.

The limitations of our study include the cross-sectional nature of the data, which limits our ability to draw any causal conclusions. Only anthropometry was used to assess malnutrition; other direct methods like clinical examination and dietary assessment were not used. Indirect methods like biochemical and laboratory methods were not used because of time and monetary constraints. HFIAS also cannot identify vulnerable members. It is an ecological fallacy that means that a household that is food insecure does not imply that all its members are food insecure; hence, the results should be interpreted with caution. The accessibility of food was not determined in this study.

## Conclusions

The current study shows that the majority of the households were food secure; minimal households were mild and moderately food insecure, and none of the households had severe food insecurity. Wasting, stunting, and underweight were more common in girls. There was a significant association between wasting and household food insecurity. There was a significant association between stunting and household food insecurity. There was a statistically significant correlation between the age of the children and stunting, gender and stunting, gender and underweight, and religion and underweight. The stunting rate was significantly higher in moderately food-insecure households. There is no significant association between socioeconomic status and household food insecurity, socioeconomic status, or undernutrition. There was a significant correlation between the gender of the children and moderate food insecurity. There was a positive correlation between socioeconomic status and food-secure households. There was a negative correlation between underweight and food-secure households, stunting and food-secure households, and wasting and food-secure households. There was a positive correlation between underweight and moderate food insecurity, stunting and moderate food insecurity, and wasting and moderate food insecurity. The study recommends regular monitoring and analysis of food insecurity and malnutrition among children and women at national, state, and regional levels. Inter-sectoral coordination and the involvement of non-governmental organizations to address childhood nutrition and various causes of malnutrition are essential. Ensuring women’s empowerment is another strategy to combat household food insecurity. Creating awareness among mothers regarding child nutrition and factors influencing malnutrition, as well as awareness regarding the public food distribution system, is necessary.
